# Advanced Solid State Nano-Electrochemical Sensors and System for Agri 4.0 Applications

**DOI:** 10.3390/s21093149

**Published:** 2021-05-01

**Authors:** Ian Seymour, Tarun Narayan, Niamh Creedon, Kathleen Kennedy, Aidan Murphy, Riona Sayers, Emer Kennedy, Ivan O’Connell, James F. Rohan, Alan O’Riordan

**Affiliations:** 1 Nanotechnology Group, Tyndall National Institute, T12 R5CP Cork, Ireland; ian.seymour@tyndall.ie (I.S.); tarun.narayan@tyndall.ie (T.N.); creedon.niamh@gmail.com (N.C.); kathleen.kennedy@tyndall.ie (K.K.); 2 Microelectronics Circuit Centre Ireland, Tyndall National Institute, T12 R5CP Cork, Ireland; aidan.murphy@mcci.ie (A.M.); ivan.oconnell@mcci.ie (I.O.); 3 Teagasc Food Research Centre Moorepark, Fermoy, P61 C996 Cork, Ireland; riona.sayers@gmail.com (R.S.); emer.kennedy@teagasc.ie (E.K.); 4 Electrochemical Materials and Energy Group, Tyndall National Institute, T12 R5CP Cork, Ireland; james.rohan@tyndall.ie

**Keywords:** electrochemical sensors, agriculture, nanosensors, biosensors, nitrates, pesticides, pH control, virus detection

## Abstract

Global food production needs to increase in order to meet the demands of an ever growing global population. As resources are finite, the most feasible way to meet this demand is to minimize losses and improve efficiency. Regular monitoring of factors like animal health, soil and water quality for example, can ensure that the resources are being used to their maximum efficiency. Existing monitoring techniques however have limitations, such as portability, turnaround time and requirement for additional reagents. In this work, we explore the use of micro- and nano-scale electrode devices, for the development of an electrochemical sensing platform to digitalize a wide range of applications within the agri-food sector. With this platform, we demonstrate the direct electrochemical detection of pesticides, specifically clothianidin and imidacloprid, with detection limits of 0.22 ng/mL and 2.14 ng/mL respectively, and nitrates with a detection limit of 0.2 µM. In addition, interdigitated electrode structures also enable an in-situ pH control technique to mitigate pH as an interference and modify analyte response. This technique is applied to the analysis of monochloramine, a common water disinfectant. Concerning biosensing, the sensors are modified with bio-molecular probes for the detection of both bovine viral diarrhea virus species and antibodies, over a range of 1 ng/mL to 10 µg/mL. Finally, a portable analogue front end electronic reader is developed to allow portable sensing, with control and readout undertaken using a smart phone application. Finally, the sensor chip platform is integrated with these electronics to provide a fully functional end-to-end smart sensor system compatible with emerging Agri-Food digital decision support tools.

## 1. Introduction

With the global population continuing to expand, there is now an urgent need to produce more food, up to 70% by 2050, more efficiently with the available existing finite resources [1]. This is a key societal challenge that is a call to action for global political leaders. To this end, the World Government Summit published, in 2018, their report called “*Agriculture 4.0—The Future of Farming Technology*”, which addressed the four main developments they identified as placing pressure on agriculture: demographics, scarcity of natural resources, climate change, and food waste [2]. Similarly, in 2019, the European Green Deal was introduced for the European Union (EU) and its citizens. This is “*a new growth strategy that aims to transform the EU into a fair and prosperous society, with a modern, resource-efficient and competitive economy where there are no net emissions of greenhouse gases in 2050 and where economic growth is decoupled from resource use*” [3]. Producers and processers on the front line are key to managing the transition to more sustainable systems of food production to reduce air, water and soil pollution, biodiversity loss and climate change, and excessive consumption of natural resources. New digital technologies are therefore required, at all stages of production, to enhance food security, reduce losses, increase sustainable production (through precision agriculture), increase economic return while also protecting biodiversity. Agriculture 4.0 is the term for this challenge facing the agri-food industry, and will necessarily include a greater focus on, the use of the internet of things (IoT) and big data to drive greater business efficiencies.

Digital technologies have the potential to revolutionize the whole agri-food and environment supply chain and will play a great role in transforming the traditional based agriculture industry to a knowledge based one. The increased connectivity of a global agriculture would enable producers to use their data to apply the 4R farm management strategy (Right product, Right place, Right time, Right rate), while processors can employ data to reduces losses, maximize production, increase quality and address consumer concerns, e.g., traceability. To enable this paradigm shift, new decision support tools and advanced analytics based on embedded and connected point-of-use sensors and systems are required to yield (quasi)real time data that will provide informed decision making capacity to stakeholders. Given the huge diversity in potential uses-cases, for example, plant and animal health monitoring [4,5,6]. to pesticide and antibiotics residue detection [7,8,9], through to soil and water quality measurements [10,11,12,13] a suite of new connected sensor systems that are fit for purpose are proposed. To date a number of sensors based on optical [14,15,16], chemical [17,18] and electromechanical detection mechanisms [19,20] have been developed, by a number of research groups, but can be limited in terms of portability, deployment incompatibilities and cost.

By contrast, electrochemical sensors have a number of adventitious properties that make them particularly suitable for point-of-use applications. They are highly sensitive and portable, are relatively low cost, exhibit a rapid time-to-result, are highly versatile e.g., different, electrode materials (C, Au, Pt) are suitable for a variety of different chemical molecules, and may be modified with a large variety of biomolecules for bio-sensing applications [21,22,23,24]. Furthermore, they are low power and provide an electrical transduction signal, which may be fed directly into electronic systems (without conversion) amplified, undergo edge analytics with the resultant data sent to the cloud [25,26]. Further advantages may be accrued when nanoscale or ultra-microelectrodes (typically smaller than the diffusion zone) are employed as the sensing element. These include: greater sensitivity arising from enhanced analyte mass transport due to radial diffusion profiles, faster analysis, lower impact of solution resistance reducing/eliminating the need for background electrolyte and greatly reduced capacitive charging [27,28,29]. Consequently, electrochemical nanosensors have tremendous potential for use in sustainable agri-Food and environmental point-of-use deployment applications.

In recent years, advances in fabrication techniques have enabled the development of solid-state micro/nano electrochemical sensor devices [28,30,31,32]. Using these fabrication approaches, we are developing a highly versatile silicon chip-based electrochemical sensor platform that incorporates multiple sensing electrodes, on-chip counter and reference electrodes, with electrical connection of the sensors to custom electronics facilitated through edge connectors based on SD card pinouts. This sensor platform is highly versatile, may be easily integrated with analogue front end electronics and can address a number of sensing challenges described above. Consequently, in this paper we present: (i) highly sensitive detection of neonicotinoid insecticides (~1 part per billion) in water using gold nanowire arrays, (ii) copper modified sensors for selective nitrate detection (1–100 µmol/L), (iii) bio-modified sensors for bovine viral diarrhoea disease detection (both viruses and antibodies), (vi) we demonstrate generator-collector electrochemical approaches for both pH measurements and in-situ pH control, required for remote analysis. Finally, (v) we integrate these sensor devices with bespoke electronics controlled using a custom written app on a smart phone and thus demonstrate an end-to-end sensor system.

## 2. Materials and Methods

### 2.1. Nanowire Fabrication

Silicon chip based devices were fabricated using methods similar to those described by Dawson et al. [33,34]. Chips were designed to interface with external electronics via a microSD port to facilitate facile electrical connection. Gold nanowire array electrodes are fabricated using a hybrid electron beam/photolithography process on a four-inch wafer silicon substrates bearing a 300 nm layer of thermally grown silicon dioxide. Blanket metal evaporations of titanium (10 nm) and gold (100 nm) using a FC-2000 E-beam evaporator (Scotech, Refrewshire, Scotland) and lift-off technique yields single, and arrays of nanowires. A second metal evaporation and lift-off process yields the interconnection tracks, contact pads and the gold counter electrode (90 µm × 7 mm). Finally, a third metal evaporation was performed to create the platinum pseudo reference electrode. To prevent unwanted interactions along the connection tracks, silicon nitride, which acts as an insulating layer was deposited by plasma, enhanced chemical vapour deposition. Photolithography and dry etching were utilized to selectively open windows (45 μm × 100 μm) in the insulating SiN layer over the microband electrodes for exposure to the electrolyte. Openings were also created over the counter and pseudo-reference electrodes and the contact pads. Each device contains six interdigitated electrode (sensors) which are separated by 0.94 mm. Once the sensor fabrication is completed, a wafer was diced into 28 separate chip devices.

### 2.2. Ultramicro Electrode Array Fabrication

Single microbands and interdigitated arrays were fabricated as in the previous section, with the initial lithography step being photolithography rather than e-beam lithography. Each chip consisted of two combs of gold working interdigitated electrodes (55 µm × 1 µm × 60 nm), platinum pseudo reference and gold counter electrode. The interdigitated structures had gaps between the combs of 2 µm.

### 2.3. Optical Characterisation

Optical micrographs were acquired using a calibrated microscope (Axioskop II, Carl Zeiss Ltd., Cambridge, UK) equipped with a charge-coupled detector camera (CCD; DEI-750, Optronics, Muskogee, OK, USA). Scanning electron microscopy analysis was undertaken to characterize the substrate surface after the fabrication procedure. SEM images were acquired using a calibrated field emission SEM (JSM-7500F, JEOL UK Ltd., Welwyn Garden City, UK) operating at beam voltages between 3 and 5 kV.

### 2.4. Electrochemical Characterization for Nanowire Arrays

All electrochemical experiments were performed using a bipotentiostat (MAC80150 Autolab, Utrecht, The Netherlands) and a Faraday cage connected to a PC. A three electrode electrochemical system was implemented; using a single microband (1 µm wide, 45 µm long) or an array of 4 gold nanowires (100 nm wide, 45 µm long, separation 500 nm) as the working electrodes versus the on-chip platinum pseudo-reference and gold counter electrodes. The cell incorporates a custom chip holder and microSD edge connector to permit electrical contact to the working electrodes, and a sample well for the electrolyte solution. Prior to all electrochemical measurements, electrodes were cleaned using a mixed solvent clean process (acetone, isopropyl alcohol and DI water) for 15 min and dried under a stream of nitrogen. To confirm electrode functionality cyclic voltammetry (CV) experiments were first undertaken in a 1 mM Ferrocene monocarboxylic acid (FCA, Sigma Aldrich, Wicklow, Ireland) solution in 10 mM phosphate buffer saline solution (PBS, pH 7.4, Sigma Aldrich). CV measurements were carried out in the potential range −0.2 to 0.6 V at a scan rate of 100 mVs^−1^.

### 2.5. Electrochemical Characterization for Interdigitated Arrays

Each chip was inspected using optical microscopy to identify any obvious defects or faults. Prior to any electrochemical characterization, chips were cleaned by immersion in acetone, then isopropyl alcohol and finally de-ionized water, each for a period of ten minutes. The chips were dried in a flow of nitrogen and placed in the chip holder. Electrochemical analysis was performed using a BA Module (Metrohm, Utrecht, The Netherlands). CVs were performed from 0 V to 0.6 V at 50 mV/s in 1 mM FCA. During these scans, the second interdigitated comb of electrodes were held at 0 V. All electrochemical measurements were recorded versus a saturated calomel electrode (SCE).

### 2.6. Electrochemical Analysis of Neonicotinoids

Imidacloprid and clothianidin were purchased from Sigma-Aldrich and used as received. Acetate buffer was purchased from Sierra Sensors (Hamburg, Germany). Square wave voltammetry (SWV) was performed by sweeping the potential from −0.2 V to −1.4 V in acetate buffer. All scans were performed using a frequency of 10 Hz, amplitude of 50 mV and a potential step of 4 mV. Blank SWV of acetate buffer (pH 5) were also obtained for the purpose of background subtraction. Imidacloprid and clothianidin stock solutions (in methanol–DI water, 1:1) were prepared at different concentrations, and were added sequentially to 90 μL AB in the cell using a serial addition approach. Five replicate SWV scans were undertaken for each addition. All experiments were performed at room temperature.

### 2.7. Electrochemical Reduction of Nitrate

SWV was also used for the electro-analysis of nitrate. First, copper was deposited onto a platinum electrode to facilitate selective nitrate detection. The deposition was done using chronoamperometry in an electrolyte of copper sulphate with boric acid (pH 2). A constant voltage of −0.3 V was applied to the working electrode versus the reference electrode for 300 s. The electrochemical studies were performed in 0.1 M Na_2_SO_4_ buffer adjusted to pH 2 using H_2_SO_4_. Different nitrate concentration (1–100 mM) were made using sodium nitrate (NaNO_3_) additions. The SWV was undertaken at a working electrode in the range of −0.4 to −1 V, with a scan rate of 0.01 V s^−1^ with a frequency of 20 Hz and modulation amplitude of 0.02 V.

### 2.8. pH Control at µIDE Arrays

A series of buffers of differing pH was used to study the gold oxide reduction reaction. 0.1 M citric acid (Riedel-de Haën, Seelze, Germany, 99.5% anhydrous) and 0.2 M sodium phosphate dibasic (Merck, Darmstadt, Germany, 99% anhydrous) were mixed in appropriate ratios to yield buffers with pH values of 3.6, 4.6 and 7.6, respectively. 0.2 M sodium phosphate dibasic and 0.2 M sodium phosphate monobasic (Sigma Aldrich, 99%) were mixed to make a pH 8.6 buffer, while 0.1 M sodium carbonate (Sigma Aldrich, 99%) and 0.1 M sodium bicarbonate (Sigma Aldrich, 99.5%) were mixed to yield pH 9 and 10 buffers. Voltammetric analysis was performed in each buffer over the potential range 0 to 1.4 V (versus Ag/AgCl) at 50 mV/s. The potential range was adjusted to account for pH variability. The pH control method was tested in a sample of 1 mM sodium bicarbonate in DI water. Generation of acidic conditions was achieved by biasing the protonator electrode at 1.8 V vs. Ag/AgCl, basic conditions were generated by biasing the protonator at −1.3 V vs. Ag/AgCl.

Stock solutions of 200 parts per million (ppm) monochloramine (MCA) were prepared and diluted with the relevant matrix to make MCA working samples. The MCA stock solution was prepared by slowly mixing a 1:1 ratio of sodium hypochlorite (NaOCl, 5% Milton Sterilizing Fluid) and ammonium chloride (NH_4_Cl, Sigma Aldrich >99%). The NaOCl was prepared by diluting a 5% bleach solution in DI water and adjusting to pH 8.3 with 1 M NaOH. The NH_4_Cl was dissolved in DI water and adjusted to pH 8.3 using 1 M HCl. The slow mixing ensures that NH_3_ is in excess, which promoted the formation of MCA. The solution was then let sit for 5 min to ensure that the reaction was complete. Electrochemical analysis of MCA solutions via pH control was undertaken. The starting potential was reduced to 0.95 V for later work. The protonator electrode was held at 1.75 V. Working samples were made by diluting the MCA stock solution with DI water. In this case, a 5 (ppm) sample was used for analysis.

### 2.9. Electrode Functionalization for Biosensor Application

CV was employed for electropolymerization deposition of *o*-aminobenzoic acid (o-ABA, 50 mM in 0.5 M H_2_SO_4_) to create a carboxylic terminated polymer layer at a gold electrode surface. Following polymerization, the electrodes were rinsed with DI water to remove any remaining monomer solution. A fresh mixture of 1:1 EDC/NHS (75 mg/mL EDC and 11.5 mg/mL NHS) was deposited onto a chip for 20 min to activate the carboxylic acid surface. Working electrodes were coated with capture biomolecules and allowed incubate for 1 h at 4 °C to allow covalent attachment to the electrode surface. Following this immobilization, the electrodes were rinsed well with acetate buffer solution containing 0.1% Tween-20 (AB-T) and DI water to remove any unbound capture biomolecules. The un-reacted active sites were blocked by immersing in 1 M ethanolamine HCl, pH 8.5 for 20 min. Impedance measurement undertaken following this step were considered as the “baseline” for the on-chip sensors

### 2.10. Detection of BVD Antibodies and Virus

For virus detection, electrodes were modified using BVD monoclonal antibody, specific to Erns antigen, as the capture biomolecule (100 µg/mL, acetate buffer, pH 4); incubated for 1 h at 4 °C. Employing antibodies to detect the Erns protein requires minimal processing as Erns is secreted from infected cells during virus replication at an adequate concentration to be used for testing serum [35,36]. BVDV in HBS-EP buffer, prepared using target Erns viral antigen (as purchased stock sample) diluted into working solutions of varying dilution (1:10 to 1:1000, respectively) using HBS-EP buffer.

For antibody detection, electrodes were modified using with BVD Virus-1 Erns antigen as the capture biomolecule. A concentration of 100 µg/mL (acetate buffer, pH 4) was prepared, deposited and allowed incubate for 1 h at 4 °C. BVDAb detection was then investigated in solutions of increasing biological complexity. BVDAb in HBS-EP buffer, prepared using BVD monoclonal antibody (RAE0823 as purchased stock sample) diluted into working solutions of varying dilution (1:10 to 1:1000, respectively) using HBS-EP buffer.

To undertake analysis, as-modified electrodes were exposed to target solutions BVD antibody or virus, initially in buffer then in serum, by spotting with 2 µL aliquots followed by incubation for 15 min at room temperature. Electrodes were again rinsed thoroughly with HBS-EP buffer and DI water to remove non-specifically bound target biomolecules prior to subsequent electrochemical measurement. All data were fitted using an equivalent circuit using NOVA software and experimental data were background subtracted using the values for the ethanolamine baseline as defined. Serological assays were benchmarked against ELISA and color coded: red—seropositive and green—seronegative.

### 2.11. Integation with Electronics

A bespoke analogue front end for interfacing to ultra-micro scale electrochemical sensors was developed, as described by Murphy et al. [25]. This was achieved by utilizing an inexpensive COTS potentiostatic system on chip, the ADuCM350 [37]. Utilizing a system on a chip dramatically reduced the cost of the system as discrete components were not required to realize the potentiostat. A digital to analog controller, the AD5683R was also used to enable an interface to dual electrode electrochemical sensors [38]. Voltammetric tests such as cyclic, square wave and generator collector voltammetry can be performed by the device. The system can be interfaced to a smartphone through Bluetooth. Test parameters can be easily programmed through a smartphone application and the results can be saved as a text file to the cloud. It is battery powered and of suitable form factor for point of sample electrochemical measurements.

## 3. Results

### 3.1. Electrode Array Characterization

**Figure 1:**
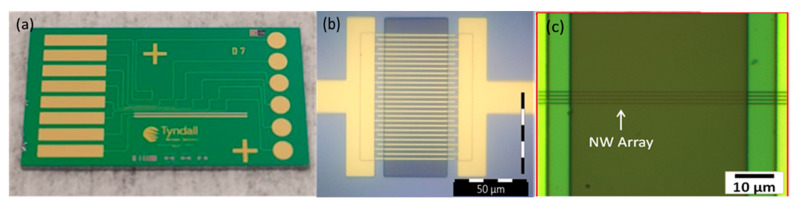
(**a**) Image of the fully fabricated chip with 6 sensors, counter and reference electrodes. (**b**) Higher magnification image of one IDE sensor. (**c**) Higher magnification image of one nanowire array.

Electrode arrays were fabricated as described in the experimental section above. Each chip contains six gold working electrodes, along with single Pt reference and Au counter electrodes. The devices were characterized using optical microscopy. Figure 1a shows an optical micrograph of the fully fabricated chip device containing the six electrically isolated electrode arrays with the Au counter and Pt reference electrodes respectively. Figure 1b shows an optical micrograph of a typical IDE sensor with a 2 µm gap between electrode combs. The protonator comb (left hand side) contains 14 electrodes, while the sensor comb (right hand side) has 13 electrodes. Figure 1c shows an optical micrograph of a typical array with four nanowire electrodes 100 nm wide and separated by 500 nm. The width of the passivation window (central dark rectangle) defined the exposed electrode length at 45 μm for both arrays.

**Figure 2:**
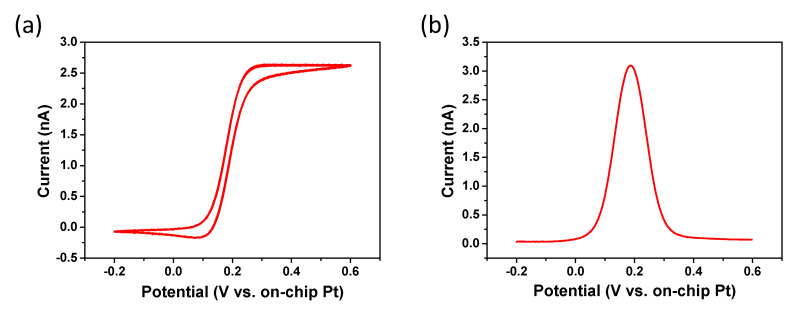
(**a**) CV and (**b**) SWV of 1 mM FCA in 10 mM PBS, pH 7.4, at 100 mV s^−1^ vs. on-chip Pt pseudo-reference electrode; illustrating the steady-state behavior of a 4 × 100 nm nanowire array separated by a distance of 500 nm.

Nanowire electrodes were structurally characterized in detail previously, by Barry et al., using a combination of optical, electron and atomic force microscopy and electrically characterized using two point current voltage measurements [39]. Furthermore, CV characterization was performed by applying a potential range of −0.2 V to 0.6 V to the nanowire arrays in 1 mM FCA in 10 mM PBS, pH 7.4, at 100 mV s^−1^; see Figure 2a. The magnitude of the current (~2.5 nA) is typical of a nano-electrode array and exhibits steady-state behaviour, as expected. This confirms that the silicon nitride passivation layer has been removed to expose the gold nanowire array and successfully shields the rest of the chip from unwanted electrochemical reactions. Only chips that exhibit this current behavior in FCA were used for experiments; chips that demonstrated low or no electrochemical current were discarded and not used for further experiments. Figure 2b shows a typical SWV of 1 mM FCA in 10 mM PBS, pH 7.4 (frequency 10 Hz, amplitude 50 mV and a potential step 4 mV) displaying a current peak in the voltage range of 0.15 to 0.25 V vs. the on-chip Pt pseudo-reference electrode. This oxidation peak is in agreement with the CV data, and further validated the oxidation of FCA is occurring at the electrodes.

**Figure 3:**
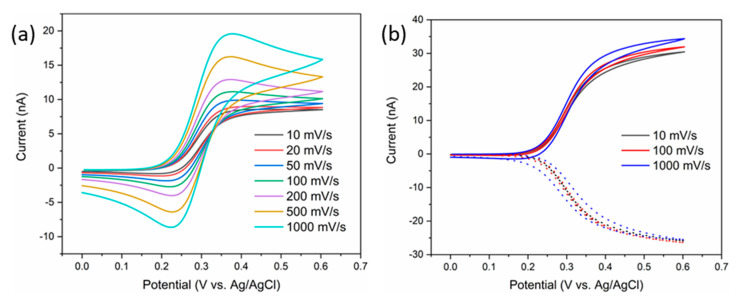
CVs of 1 mM FCA in 10 mM PBS, pH 7.4, at various scans rates with the collector (**a**) unbiased and (**b**) biased at 0 V vs. Ag/AgCl. The dotted curve in (**b**) represents the collector response to the CV at the generator electrode.

Similarly, the 2 µm gap IDE arrays were characterized in FCA, initially with no bias at the collector electrodes. As the arrays consist of micron scale electrodes separated by gaps of the same scale, diffusional overlap was expected. Figure 3a shows the effect that varying the scan rate had on the IDE array. The slower scan rates exhibited a quasi-steady state behavior, displaying a plateau rather than a peak shape. As the scan rate was increased, the behavior changed significantly, exhibited transient peaks were observed rather than steady-state plateaus. This is indicative of the arrays behaving like one larger electrode with linear analyte diffusion due to radial diffusion field overlap, rather than multiple smaller electrodes. Each IDE was then further characterized in generator-collector mode by biasing a collector electrode at a fixed potential and scanning the generator at a varied scan rate. Figure 3b shows the result of biasing the collector electrode at 0 V while scanning the generator at 10, 100 and 1000 mV/s. In GC mode, the oxidized FCA species produced at the generator diffuses across to the collector where it is subsequently reduced. This has two significant benefits. Firstly, there is no diffusional overlap as the collector prevents this from happening, resulting in the array behaving as desired. Secondly, The FCA is redox cycled between the two combs of electrodes amplifying the signal response. The scan rate is shown to have very little impact for the GC scans, as each electrode is acting independently.

### 3.2. Neonicotinoid Detection at Nanowire Arrays

**Figure 4:**
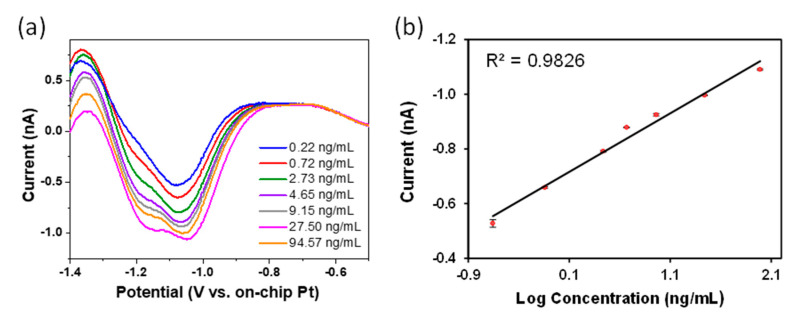
(**a**) Square wave voltammogram of Clothianidin recorded at different concentrations in acetate buffer (pH 5) versus on-chip Pt pseudo reference electrode. (**b**) Calibration plot of peak current vs. log concentration of clothianidin solution. Error bars are included in clear data points.

Following electrochemical characterization, nanowire arrays were applied to the detection of the neonicitinoid pesticides clothianidin and imidacloprid. The voltametric reduction signals observed for these compounds arise from the reduction of NO_2_ groups to NH_2_ groups, via a two- step electro-reduction pathway, which is well documented [40,41,42,43]. Firstly, a nitro intermediate followed by a hydroxylamine intermediate are formed via a 4e^−^/4H^+^ electron and proton transfer system. The hydroxylamine group is further reduced to an amine via a 2e^−^/2H^+^ electron and proton transfer system. Compared to CV, SWV is a very sensitive electrochemical method that permits fast scan rates, significantly reduces background noise and as such is suitable for remote electro-analysis. Combining this approach with the sensitivity of nanowire electrodes permits detection in simple acetate buffer thereby obviating the requirement for more complex mixed acid solutions such as Britton-Robinson buffer [41,44,45]. It was demonstrated previously that pH 4 to 9 is optimum for imidacloprid determination [46]. On this basis a pH of 5 was chosen for imidacloprid determination. Figure 4a shows typical SWV following background subtraction for clothianidin in the concentration range of 0.88–378 nM (0.22–94.57 ng/mL). The reduction peaks at −1.07 V and −1.18 V represent the two-step reduction of the nitro group of imidacloprid; supporting the reduction mechanism for nitro group reduction. The first reduction is attributed to the four electron transfer of the nitro group to yield the corresponding hydroxylamine derivative. The second reduction peak ~−1.18 V is attributed to the two electron transfer from the hydroxylamine to produce its amine derivative [47,48]. To demonstrate the suitability of the nanowire arrays as sensors for clothianidin, calibration experiments were undertaken to examine the effects of increasing concentration on the SWV signals using a serial addition approach. Figure 4b shows the calibration plot obtained by plotting the peak height at ~−1.07 V versus log of concentration. The measured detection limit for clothianidin was 0.22 ng/mL (0.88 nM) and a calibration coefficient of R^2^ = 0.984 shows good linearity with increasing concentration in this concentration range.

**Figure 5:**
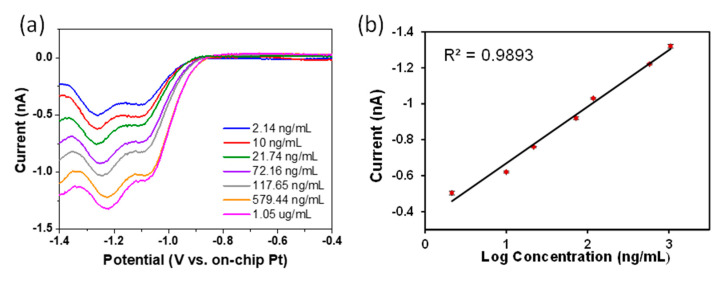
(**a**) SWV of Imidacloprid recorded at different concentrations in acetate buffer (pH 5) versus on-chip Pt pseudo reference electrode. (**b**) Calibration curve of peak current vs. log concentration of imidacloprid. Error bars are included in clear data points.

Similarly, Figure 5a shows the SWV of imidacloprid in the concentration range of 8–4.1 µM (2.15–1.05 µg/mL) following background subtraction. The CV again shows the two reduction peaks representing the nitro group reduction of imidacloprid with the peaks at ~−1.05 and −1.2 V being attributed to the first and second reduction steps, respectively. The quantitative determination of imidacloprid at gold electrodes is based on the semi-log relationship between peak current intensity (nA) and imidacloprid concentration. Figure 5b shows the calibration plot obtained by plotting the peak current at approximately –1.2 V versus log of concentration. Again, each measurement was undertaken five times to monitor the stability and reproducibility of the electrodes and the error bars represent one standard deviation from the mean value of the five replicates. The measured limit of detection was 2.14 ng/mL (8 nM) and a coefficient of R^2^ = 0.989 shows good linearity with increasing concentration in this concentration range. This concentration range is lower than the lowest legal residue limit of ~10 ng/mL for food products thus demonstrating the suitability of this technique [49].

### 3.3. Electrochemical Detection of Nitrates

**Figure 6:**
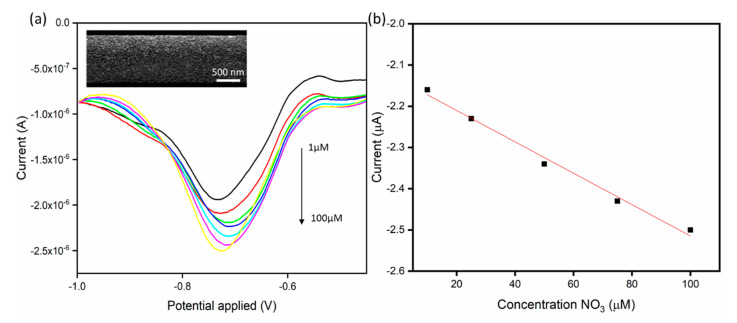
(**a**) SWV in increasing concentrations of NaNO_3_ in 0.1 M Na_2_SO_4_ at pH 2. SWV were performed from −0.4 to −1 V at 10 mV/s with an amplitude of 20 mV and a frequency of 20 Hz. A SEM image of the copper modified electrode is shown in the top inset. (**b**) The resultant calibration plot for the SWV measurements

An electrochemical deposition process described in the experimental section was used to deposit copper nanostructures on the surface of the platinum microband electrode, and is shown in the inset of Figure 6a. The reaction and kinetics of the copper nucleation on platinum microelectrodes could be used to explain morphology achieved during the deposition. Longer deposition times resulted in larger copper nanostructures being formed. Since the electrochemical deposition is performed in acidic pH, hydrogen evolution occurs simultaneously with the copper deposition (Equations (1) and (2)):

(1) $${\mathrm{Cu}}^{2+}+2e^{-}\;\to \mathrm{Cu}$$

(2) $$2H^{+}+2e^{-}\;\to H_{2}$$

As the deposition voltage of –0.3 V is applied, a constant stream of H_2_ is evolved, resulting in 3D nanoporous copper structures being formed on the surface of the platinum microelectrode.

SWV was used for the electro-analysis of nitrate, the results of which are shown in Figure 6a. It has been shown that under acidic conditions, nitrate can be reduced to ammonium ions according to the following equation (Equation (3)):

(3) $${\mathrm{NO}}_{3}^{-}+8e^{-}+10H^{+}\;\to {\mathrm{NH}}_{4}^{+}+3H_{2}O$$

To examine the sensor performance a linear calibration plot was obtained as seen in Figure 6b. It was obtained by increasing the concentration of NO_3_ using the standard addition method (i.e., 1, 10, 15, 25, 50 100 µM) resulted in a corresponding increase in current. On-chip reference and the counter electrode of platinum and gold was used for the measurement. The sensitivity of the electrode was at 0.038 A/µM with R^2^ = 0.99. The sensitivity of the microelectrode was found to be enhanced because the deposited copper layer shows a porous structure. The large surface area on microelectrode makes it more effective electrocatalyst in facilitating nitrate reduction than the standard macro electrode. The LOD calculated to be 0.2 µM with a linear range of 1–100 µM.

### 3.4. Electrochemical pH Measurement and In-Situ pH Control for the Conversion of Monochloramine to Dichloramine

**Figure 7:**
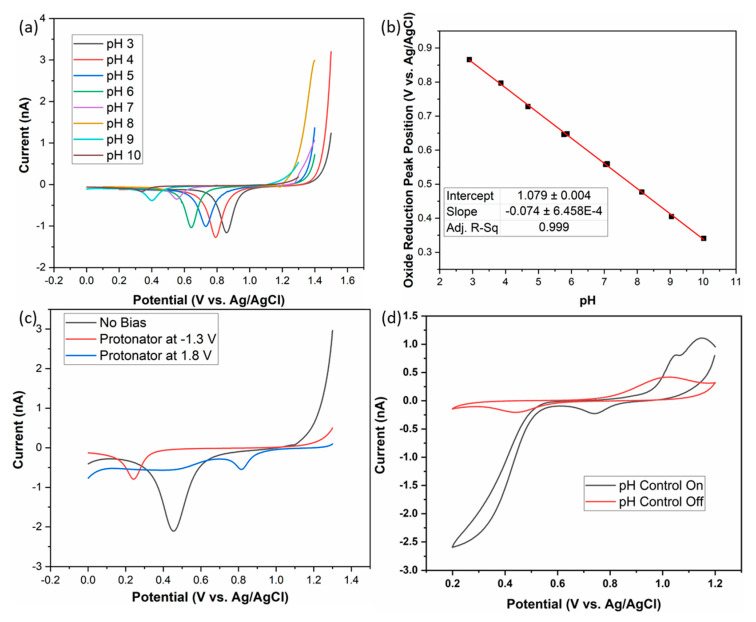
(**a**) LSVs in various pH buffers from 1.4 V to 0 V at 50 mV/s. (**b**) Calibration of the oxide reduction peak potential vs. pH. (**c**) LSVs of 1 mM Sodium Bicarbonate in DI water from 1.3 V to 0 V at 50 mV/s with various biases applied to the protonator electrode. (**d**) CVs, from 1.2 V to 0.2 V at 50 mV/s, in a 5 ppm sample of monochloramine with and without a potential bias of 1.75 V at the protonator electrode.

The gold oxide reduction peak was used as a probe for the pH condition of the electrodes [50,51]. An oxide was formed on a gold electrode by scanning to a sufficiently positive potential, typically around 1.2 V. The electrode was then swept cathodically to the initial potential and the position of the oxide reduction peak was noted. This procedure was repeated at different pH values in a series of buffers to establish the linearity of the response. Figure 7a shows the oxide reduction peaks for the different buffer solutions. In each case, four replicate scans were performed to determine reproducibility. As expected, the oxide peaks shifted to more positive potentials at lower pH values. The calibration plot, shown in Figure 7b, indicates a strong linearity with an R^2^ = 0.999. The oxide approach to pH analysis shows a sensitivity of 74 mV/pH, indicated by the slope of the linear fit. The pH control experiments are shown in Figure 7c. The initial scan, where no protonator bias was applied, indicates that the sodium bicarbonate sample had an initial pH of approximately pH 8. This was established based on the position of the oxide reduction peak at 0.45 V, which correlates to pH 8 using the calibration plot (Figure 7b). With the application of 1.8 V potential bias at the protonator, local acidic conditions were created. The potential of 1.8 V is in the Oxygen evolution region; thus an excess of protons was produced from the water splitting. Therefore, the sensor electrode was exposed to conditions that were more acidic than the bulk environment, in this case it was approximated to be pH 3, as above. Similarly, more basic conditions could be created by applying a potential of −1.3 V at the protonator electrode. With this potential bias, hydrogen gas was produced and an excess of hydroxyl were produced in the vicinity of the sensor electrode. The position of the oxide reduction peak indicated a pH environment outside of the calibration range, but assuming linearity, was approximated to be pH 11.5.

The acidic pH control method was subsequently applied to the conversion of monochloramine to dichloramine. At pH 8 and above, chloramines tend to exist predominantly as monochloramine. As sample pH becomes more acidic, this monochloramine is converted to dichloramine. This conversion can be monitored electrochemically as dichloramine shows electroreduction at potentials more anodic than the reduction of monochloramine. Figure 7d shows the result of applying the pH control method to a 5 ppm sample of monochloramine. Without a protonator bias, no reduction event was observed attributable to the chloramine species. The oxide reduction peak also indicated a pH environment of approximately pH 8. By imposing a 1.75 V bias at the protonator, acidic conditions were generated, indicated by the shift in the oxide reduction peak potential. The anodic shift indicated that the local environment was significantly more acidic than the initial conditions. A reduction event with an onset potential of ~0.5 V was also observed, which was attributed to the reduction of dichloramine, confirming the conversion of monochloramine to the new species. There were two major advantages to applying this technique to the detection of monochloramine. The first was that variability of pH was eliminated. In real water samples, pH conditions can vary dramatically as a result of numerous environmental factors. As such, this introduces complications, particularly if the species is pH sensitive. The second advantage was that the conversion of monochloramine to dichloramine eliminated oxygen as an interfering species. The reduction potential of monochloramine overlaps with the reduction of dissolved oxygen, leading to convoluted signals. Dissolved oxygen is common at various concentrations in water, therefore it cannot be easily background subtracted. The reduction of dichloramine however was found to be free of oxygen interference, thus simplifying its quantification.

### 3.5. Label-Free Immunoassays

**Figure 8:**
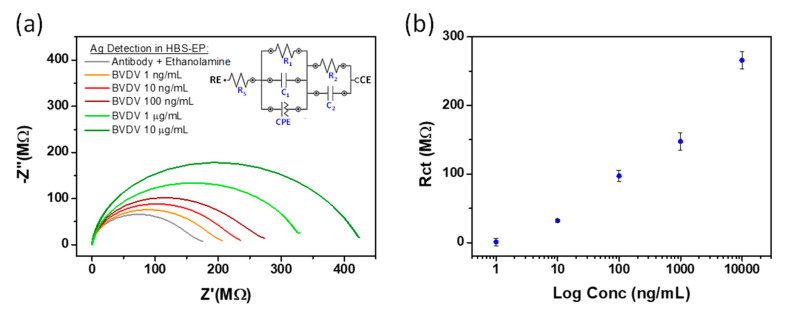
(**a**) Nyquist plots of 1mM FcCOOH showing quantitative detection of recombinant BVDV1 Erns protein in HBS-EP buffer. (**b**) Semi-log relationship of the charge transfer resistance versus virus concentration (error bars represent *n* = 3 replicates) background subtracted using ethanolamine baseline.

With regard to label free immunoassays at the electrochemical sensing platform, initial development was undertaken by spiking know concentrations of target analyte into HBS-EP. Figure 8a shows the Nyquist diagrams, in the presence of 1 mM FcCOOH, measured an electrode surface following modification with antibody and exposure to different concentration of viral protein in HBS-EP buffer ranging from 1 ng/mL to 10 µg/mL. A corresponding increase in measured Rct (~200 MΩ to ~425 MΩ) was observed. This increase can be attributed to the virus binding as it acts as a kinetic barrier for electron transfer. These results also suggest that the antibody specificity/functionality is not hindered by its covalent attachment to an electrode. A modified equivalent circuit, to account for the additional electrode bio-layers, was used to fit the results. Additional resistive and capacitive elements have been added where R1 and R2 both represent the charge transfer resistance, while C1 and C2 represent the double layer and coating capacitances respectively. All the capacitances shown in the equivalent electrical circuit are mathematically modelled using a constant phase element (CPE) in NOVA software; and represent all the frequency dependent electrochemical phenomena. Figure 8b shows the semi-log linear relationship between the Rct sensor response and virus concentration thereby suggesting viral detection is possible with high sensitivity and a large dynamic range (1 ng/mL to 10 µg/mL). The error bars represent n = 3 replicates. Similar results were obtained for BVD Virus-1 Erns antigen (as the capture biomolecule) modified electrodes in HBS-EP buffer with different concertation of antibodies but are not presented here for brevity.

Concerning serological label-free immunoassays, the immunosensors were applied to BVDV and BVDAb detection in whole (undiluted) serum. These experiments were undertaken to assess suitability of the sensors for use as on-farm diagnostic applications. To this end, (i) virus-modified sensors were applied to the detection of BVDAb seropositive and seronegative samples; and (ii), antibody-modified sensors were applied to the detection of BVDV in PI calves (virus positive) and virus negative samples. The electrodes were again characterized using EIS and data fit as previously described.

### 3.6. Antibody Detection in Serum

**Figure 9:**
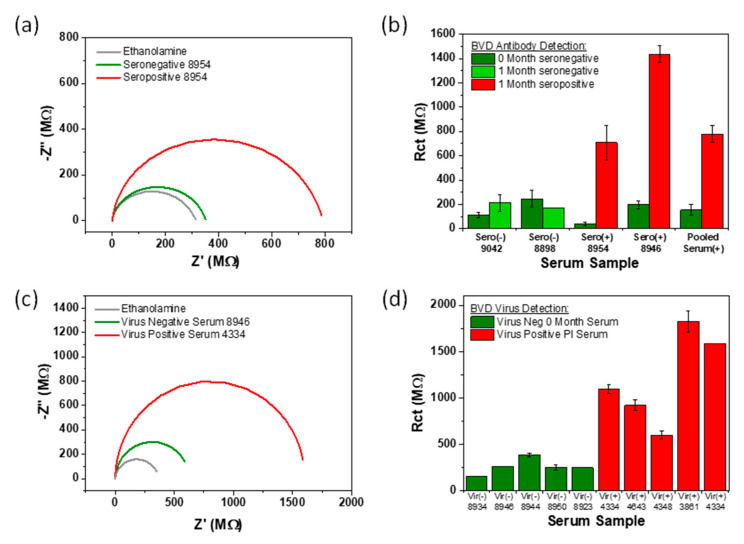
(**a**) Nyquist plots of seropositive and seronegative blood deposition on BVD virus (10 μg/mL) modified microelectrodes, in the presence of 10 mM PBS containing 1 mM FcCOOH. (**b**) Bar chart comparison of seropositive samples and their respective seronegative samples, background subtracted from their respective ethanolamine baselines. (**c**) Nyquist plots of a virus negative and virus positive PI serum, deposited on BVD antibody modified nanoband (measured in the presence of 1 mM FcCOOH). (**d**) Bar chart testing PI serum and virus negative serum samples, background subtracted from their respective ethanolamine baselines.

A number of sensor chips were modified with virus (10 µg/mL) to test for BVDAb in pooled and unpooled seropositive and seronegative samples. A spotting technique was again employed to deposit multiple sera samples on separate electrodes on a chip. Typical EIS measurements for the detection of BVDAb in a single calf (No.8954) obtained at time 0 month, and 1 month are presented in Figure 9a. Data from the seronegative sample produced an electrode impedance of ~350 MΩ, a ~30 MΩ increase compared to the baseline attributed to small amounts of non-specific binding (green plot). A significant increase in impedance was observed following incubation with a seropositive sample ~800 MΩ (red plot). This increase may be attributed to binding of the BVD antibodies present in the serum to the viral modified electrode surface. Figure 9b shows experimental background subtracted Rct EIS data in a bar chart format, obtained for a number of individual and pooled samples when undertaking BVDAb detection in seronegative and seropositive samples. Zero month samples (known to be seronegative) for an individual calf or pool are shown as dark green bars. These 0 month samples all exhibited very low impedance (<250 MΩ) for antibody detection, as expected. One month seronegative samples for two individual calves are presented as light green bars. The small difference in impedance values the zero and one month samples may be attributed to slight variation in the degree of non-specific adsorption and in electrode preparation. However, a clear increase in the electron-transfer resistance (>800 MΩ) red bars is observed between the negative controls and both individual and pooled seropositive samples, (time to results 15 min). These results strongly support the suitability of these sensors for use as on-farm diagnostic devices as the sensors can discriminate between seropositive and seronegative in undiluted blood serum.

### 3.7. Virus Detection in Serum

A number of sensor chips, modified with monoclonal BVDAb (10 µg/mL), were employed to test for the presence of BVD virus in persistently infected (PI) calf whole serum. In the same manner as previously discussed, a spotting technique was used to deposit PI serum and virus negative serum on electrodes 3–5 and 1–2, respectively. Nyquist plots, illustrating detection of BVDV positive serum (from PI calf 4334) and BVDV negative serum (from 0 month calf 8946) on the same microSD chip, are shown in Figure 9c. The deposition of BVDV negative serum 8946 shows an Rct of ~600 MΩ, which increased slightly following the ethanolamine baseline ~350 MΩ, Figure 9c green plot. As mentioned previously, this data suggests there is a small formation of a more insulating layer on the electrode which could be attributed to increasing nonspecific binding from protein in the bovine serum (like albumin) to the antibody modified electrode surface. On a separate electrode on the same chip, the target virus positive PI serum was immobilised and again, revealed a substantial Rct value of ~1600 MΩ; an increase of ~1230 MΩ from the baseline, which compensates for the small amount of non-specific binding reported from the negative serum samples. We suggest that this large increase in Rct arises from the large virus molecules acting as a kinetic barrier to the transfer of electrons from the FcCOOH to the electrode, hence increasing the system resistance.

In order to validate these findings, several more PI serum samples (confirmed by ELISA) were tested for presence of virus and compared to virus negative serum samples. In total, five BVDV negative samples (green) and five BVDV positive PI samples (red) were examined and the Rct value measured. The chart in Figure 9d shows the background subtracted Rct data for these assays. As expected, the virus negative sera exhibited minor NSB binding to the electrode (<400 MΩ), whereas the target seropositive 1 month samples present a significant increase in the electron-transfer resistance (>800 MΩ). These results propose that there is successful serological binding of the BVDAb to the immobilised viral protein. The results presented above were benchmarked, colour coded and in very good agreement with ELISA using commercial plates for BVDV and ENRS. These results clearly demonstrate that the nanoband sensors have sufficient sensitivity and specificity for detection of both target antibody and virus detection in serum. The findings are of particular significance for on-farm point of use applications where high sensitivity and specificity and low time-to-results, are required to permit early diagnostics by veterinarians.

### 3.8. System Integration

**Figure 10:**
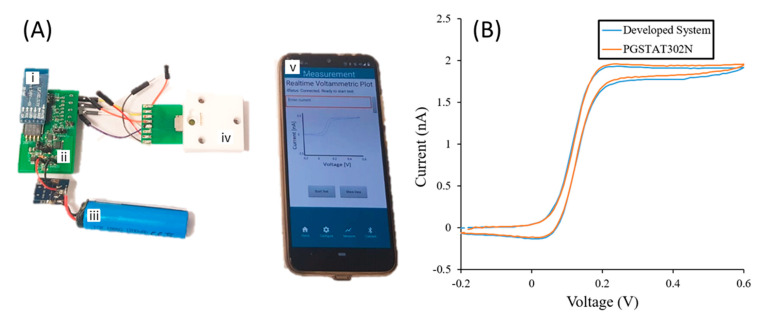
(**A**) Developed system interfacing to electrochemical sensor with (i) Bluetooth module, (ii) developed system printed circuit board, (iii) Lithium ion battery connected to charge protection circuit, (iv) electrochemical sensor holder and (v) Smartphone using developed application. (**B**) CVs at 200 mV/s for 1 mM FCA oxidation using both the developed system and the PGSTAT302N.

A bespoke electrochemical sensor system was developed, using a commercial potentiostat integrated circuit chip, that was capable of controlling the sensor platform, reading the electrochemical response and displaying these results via a smartphone app as shown in Figure 10A. The system was designed to perform CV and SWV with a 2 V window, from −1 V to +1 V and also operate in bi-potientiostat mode. The system was benchmarked against a portable laboratory potentiostat, the PGSTAT302N, to show that it is fit for purpose for ultra-micro and nano scale electrochemical sensors. A CV was performed using FCA on both systems. As seen from Figure 10B the CVs of the two potentiostats are in close agreement. A reduced steady-state current of the developed system’s voltammogram was expected as it was the second measurement taken in the calibration. A EUR 90.30 bill of materials price means that the system is more cost effective alternative to commercial portable potentiostats. Similarly, as the data is displayed by means of the smartphone app, no additional interface devices are required to achieve an analytical response.

## 4. Conclusions

In this work, we have shown the versatility of the electrochemical sensor platform by demonstrating a wide range of applications from chemical sensing to biological sensing along with system integration. With the advancement of electrode modification techniques, along with tailored electrochemical analysis, these devices can address many of the sensing requirements associated with the emerging agricultural 4.0 industry. With these devices, the detection of pesticides, fertilizer agents of infectious disease were possible on extremely short timescales. These devices can be tailored to be free of matrix interference effects by mitigating pH variability. This particular technique can promote the detection of a species of interest, or alternatively mask detection of an interfering species. Finally, we have shown a portable electrochemical reader system, capable of performing the desired analysis on farm. The significance of this is that the turnaround time between getting an analytical result and responding to any issues is at a minimum, as the two events can be completed on-site in a matter of minutes.

## Data Availability

Data is contained within the article.
